# Prevalence and associated factors of excessive daytime sleepiness in rural older adults: a population-based study

**DOI:** 10.1007/s11325-024-03004-5

**Published:** 2024-02-19

**Authors:** Juan Ren, Rui Liu, Tong Zhao, Jie Lu, Cuicui Liu, Tingting Hou, Yongxiang Wang, Lin Cong, Yifeng Du, Shi Tang, Chengxuan Qiu

**Affiliations:** 1 Department of Neurology, Shandong Provincial Hospital, Shandong University, Jinan, Shandong People’s Republic of China; 2 Department of Neurology, Shandong Provincial Hospital Affiliated to Shandong First Medical University, Jinan, Shandong People’s Republic of China; 3 Department of Ultrasound, Shandong Provincial Hospital Affiliated to Shandong First Medical University, Jinan, Shandong People’s Republic of China; 4 Shandong Provincial Clinical Research Center for Neurological Diseases, Jinan, Shandong People’s Republic of China; 5 Medical Science and Technology Innovation Center, Shandong First Medical University & Shandong Academy of Medical Sciences, Jinan, Shandong People’s Republic of China; 6 Institute of Brain Science and Brain-Inspired Research, Shandong First Medical University & Shandong Academy of Medical Sciences, Jinan, Shandong People’s Republic of China; 7 Aging Research Center and Center for Alzheimer Research, Department of Neurobiology, Care Sciences and Society, Karolinska Institutet-Stockholm University, Stockholm, Sweden

**Keywords:** Excessive daytime sleepiness, Prevalence, Rural, Older adults, Population-based study

## Abstract

**Objective:**

To investigate the prevalence and associated factors of excessive daytime sleepiness (EDS) among rural-dwelling Chinese older adults.

**Methods:**

We collected data on demographic, epidemiological, and clinical factors via in-person interviews and clinical examinations following a structured questionnaire. The 15-item Geriatric Depression Scale (GDS-15) was used to assess depressive symptoms, the Berlin questionnaire (BQ) to assess obstructive sleep apnea (OSA) risk; and the Epworth Sleepiness Scale (ESS) to assess sleep characteristics. EDS was defined as the total ESS score > 10.

**Results:**

This population-based study engaged 4845 participants (age ≥ 65 years, 57.3% female) in the 2018 examination of the Multimodal Interventions to Delay Dementia and Disability in Rural China. The prevalence of EDS was 9.3% in the total sample, 8.3% in females, and 10.6% in males, and the prevalence decreased with advanced age. Logistic regression analysis revealed that EDS was significantly associated with age (multivariable-adjusted odds ratio [OR] = 0.97; 95% confidence interval [CI] 0.95–0.99), female sex (0.53; 0.36–0.77), hypertension (0.68; 0.54–0.85), depressive symptoms (2.68; 2.07–3.46), high OSA risk (2.11; 1.69–2.63), and poor sleep quality (2.12; 1.60–2.82).

**Conclusion:**

EDS affects nearly one-tenth of rural older adults in China. Older age, female sex, and hypertension were associated with a decreased likelihood of EDS, while depressive symptoms, high OSA risk, and poor sleep quality were correlated with an elevated likelihood of EDS.

**Supplementary Information:**

The online version contains supplementary material available at 10.1007/s11325-024-03004-5.

## Introduction

Excessive daytime sleep (EDS) is defined as the inability to stay awake and alert during major waking episodes, resulting in periods of irrepressible need for sleep or unintended lapses into drowsiness or sleep [1]. EDS, as a public health concern among older adults, has been correlated with cognitive decline, poor quality of life, malnutrition, and behavioral deficits. Several studies from France and the USA have suggested that prevalence of EDS ranges from 8.6% to 40% [1–3], and factors associated with EDS included male sex, alcohol consumption, smoking, high body mass index (BMI), leisure-time physical inactivity, diabetes, coronary heart disease (CHD), stroke, and depression [1, 3, 4]. However, data on the prevalence of EDS and associated factors among elderly people in rural regions of China is still lacking.

Thus, we sought to investigate the prevalence and related factors of EDS among older Chinese rural adults in this large-scale, community-based cross-sectional study.

## Methods

### Study design and participants

This cross-sectional study utilized information from the Multimodal Interventions to Delay Dementia and Disability in Rural China (MIND-China) [5]. The MIND-China study targets residents living in Yanlou Town, Yanggu County of western Shandong Province who are at least 60 years old at the end of 2017. The interdisciplinary baseline assessments were performed before recruiting participants for interventions, as previously reported [5]. Briefly, from March to September 2018, the MIND-China baseline examinations were incorporated into the annual medical checkups provided by Yanlou Town hospital for local residents who were aged over 65 years. In addition, the MIND-China intervention study specifically invited participants aged 60 to 64 years.

The MIND-China protocol was reviewed and approved by the Ethics Committee at Shandong Provincial Hospital in Jinan, China. Each participant or a proxy signed a written informed consent form. We registered MIND-China in the Chinese Clinical Trial Registry (ID: ChiCTR1800017758).

### Data collection

Following the structured questionnaire, the trained medical staff collected data via face-to-face interviews, routine clinical examinations, neuropsychological tests, and laboratory blood test [5]. The questionnaire included information on sociodemographic characteristics, health behavior or lifestyle factors, medical history or health conditions, laboratory data, use of medications, and sleep characteristics. The detailed description of data collection and assessments was presented in the Supplementary Methods.

### Assessment of EDS

A Chinese version of the Epworth Sleepiness Scale (ESS) was used to assess daytime sleepiness. The ESS requested participants to rate eight everyday situations related to sleep on a Likert scale from hardly falling asleep to easily falling asleep, with the score ranging from 0 to 3. A total score of 0–24 is calculated from the individual item scores. An ESS score above 10 is considered to be indicative of EDS [1].

### Assessment of covariates

The presence of depressive symptoms was identified as a total score of 5 or higher on the 15-item Geriatric Depression Scale (GDS-15). The risk of obstructive sleep apnea (OSA) was assessed with the Berlin questionnaire (BQ). The BQ assessed three categories of symptoms, of which, two or more positive scores indicated a high risk of OSA; one or none indicated a low risk of OSA. Supplementary Methods provide detailed description.

### Statistical analysis

Study participants' sociodemographic characteristics, clinical conditions, and behavioral factors were presented by EDS status. Categorical variables were compared using the Chi-square test, while non-normally distributed continuous variables were compared using the Mann–Whitney *U* test. Specifically, we reported prevalence of EDS by age groups and sex. We used logistic regression models to estimate the odds ratio (OR) and 95% confidence interval (CI) for the association of EDS with various factors, while controlling for multiple potential confounding factors. The main results were reported from two models: model 1 was controlled for age, sex, and education; and model 2 was further controlled for alcohol drinking, smoking, leisure-time physical activities, BMI, hypertension, dyslipidemia, diabetes, CHD, stroke, OSA risk, presence of depressive symptoms, hypnotics use, sleep duration, and sleep quality. We considered a two-tailed *P* < 0.05 to be statistically significant. All statistical analyses were conducted using IBM SPSS Statistics for Windows, Version 26.0 (IBM Corp., Armonk, NY).

## Results

### Characteristics of study participants

In March-September 2018, a total number of 5765 participants who were aged 60 years or older undertook the multidisciplinary assessments for MIND-China [5]; of them, 519 persons who were aged 60–64 years were excluded because people in this age group were substantially underrepresented, and additional 401 were excluded due to missing data on the ESS, leaving 4845 participants for the current analysis.

Of the 4845 participants, the mean age was 70.3 years (standard deviation [SD] = 5.0) and 57.3% were female. Compared with participants without EDS, those with EDS were younger, more likely to be male, had a higher BMI, more likely to drink alcohol, less likely to take leisure-time physical activities, had a shorter sleep duration, high OSA risk, worse sleep quality, and more likely to have a history of stroke and depressive symptoms (*P* < 0.05) (see Supplementary Table 1). The two groups had no significant differences in educational level, smoking, diabetes, hypertension, dyslipidemia, CHD, and use of hypnotics (*P* > 0.05).

### Prevalence and distribution of EDS

**Fig. 1:**
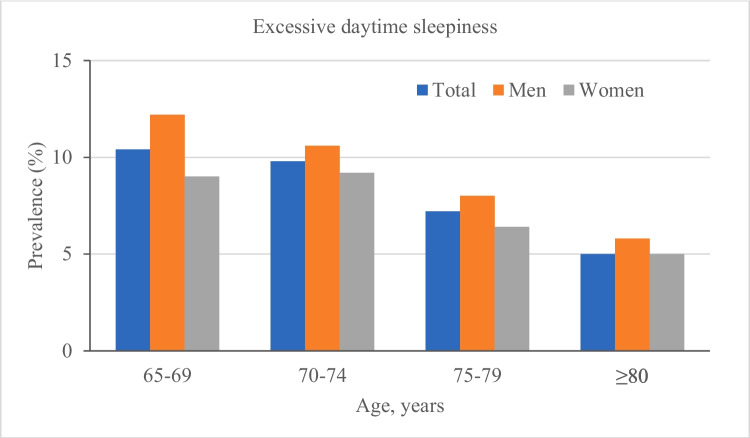
Age- and sex-specific prevalence (per 100 population) of excessive daytime sleepiness

The overall prevalence of EDS was 9.3% (95% CI: 8.5%-10.1%) in the total sample, 10.6% (9.2%-11.9%) in males, and 8.3% (7.3%-9.4%) in females (for sex difference, *P* < 0.01). Figure 1 shows the age- and sex-specific prevalence of EDS. In the total sample, the prevalence of EDS decreased with age, from 10.4% in people aged 65–69 years to 5.0% in those aged ≥ 80 years. Similarly, the prevalence of EDS decreased with age in both males and females. Moreover, the prevalence of EDS was higher in males than in females across all age groups.

### Correlates of EDS

Logistic regression analysis suggested that controlling for multiple potential confounders, older age and female sex were significantly associated with a decreased likelihood of EDS (Table 1).

**Table 1:** Demographic, lifestyle, and clinical correlates of excessive daytime sleepiness

Characteristics	No. of subjects	No. of EDS cases	Odds ratio (95% confidence interval), EDS	
			Model 1^a^	Model 2^a^
Age, years	4845	450	0.96 (0.94–0.98)^***^	0.97 (0.95–0.99)^**^
Female sex	2774	231	0.66 (0.53–0.83)^***^	0.53 (0.36–0.77)^***^
Educational level				
Illiteracy	1888	180	1.00 (reference)	1.00 (reference)
Elementary school	2154	198	1.19 (0.89–1.59)	1.07 (0.79–1.44)
Middle school or above	803	72	1.49 (1.07–2.08)^*^	1.34 (0.95–1.89)
Body mass index (kg/m^2^)	4820	446	1.03 (1.00–1.05)	1.02 (0.99–1.04)
Alcohol consumption				
Never	2970	258	1.00 (reference)	1.00 (reference)
Former	460	60	1.28 (0.88–1.88)	1.16 (0.77–1.74)
Current	1415	132	0.87 (0.64–1.18)	0.95 (0.69–1.32)
Smoking				
Never	3118	272	1.00 (reference)	1.00 (reference)
Former	999	94	0.78 (0.55–1.12)	0.80 (0.55–1.17)
Current	728	84	1.04 (0.72–1.50)	0.97 (0.66–1.43)
Leisure-time physical activities	3236	280	0.85 (0.69–1.04)	0.87 (0.70–1.07)
Hypertension	3216	286	0.90 (0.73–1.10)	0.68 (0.54–0.85)^***^
Diabetes	701	78	1.27 (0.98–1.65)	1.10 (0.83–1.45)
Dyslipidemia	1152	106	1.05 (0.84–1.33)	0.92 (0.72–1.17)
Coronary heart disease	1061	109	1.23 (0.98–1.55)	0.99 (0.78–1.26)
Stroke	765	98	1.57 (1.24–2.00)^***^	1.20 (0.92–1.55)
Depressive symptoms	506	117	3.66 (2.88–4.65)^***^	2.68 (2.07–3.46)^***^
Hypnotics	240	26	1.29 (0.84–1.96)	0.72 (0.46–1.13)
Sleep duration				
≤ 6 h	1894	202	1.43 (1.16–1.77)^***^	0.87 (0.66–1.15)
> 6 to 8 h	2244	180	1.00 (reference)	1.00 (reference)
> 8 h	674	65	1.26 (0.93–1.70)	1.28 (0.94–1.75)
OSA risk, *n* (%)				
Low	3413	237	1.00 (reference)	1.00 (reference)
High	1426	213	2.27 (1.86–2.76)^***^	2.11 (1.69–2.63)^***^
Sleep quality				
Good	3241	233	1.00 (reference)	1.00 (reference)
Poor	1571	214	2.29 (1.87–2.80)^***^	2.12 (1.60–2.82)^***^

*Abbreviations*: *EDS* excessive daytime sleepiness, *OSA* obstructive sleep apnea

^a^Model 1 was adjusted for age, sex, and education, and Model 2 was additionally adjusted for all the other factors included in the table

^*^ P < 0.05, ^**^ P < 0.01, ^***^ P < 0.001

When sociodemographic factors were controlled for, stroke history, depressive symptoms, short sleep duration, high OSA risk, and poor sleep quality were significantly associated with an increased likelihood of EDS (Table 1, model 1). In the multivariable-adjusted model, older age, female sex, and hypertension were related to a decreased likelihood of EDS, whereas the presence of depressive symptoms, high OSA risk, and poor sleep quality were correlated with an increased likelihood of EDS; the associations between stroke history and sleep duration with EDS became statistically non-significant (Table 1, model 2). Diabetes, dyslipidemia, CHD, and use of hypnotics were not significantly associated with EDS (Table 1).

## Discussion

This population-based study revealed that almost one-tenth of rural-dwelling Chinese older adults (age ≥ 65 years) suffered from EDS. Overall, the prevalence of EDS decreased with advanced age, and males had a higher prevalence of EDS than females overall and across all age groups. The presence of depressive symptoms, high OSA risk, and poor sleep quality were related to an increased prevalence of EDS, whereas hypertension was correlated with a decreased prevalence of EDS.

The overall prevalence of EDS in our sample (9.3%) was comparable to the report from the Honolulu-Asian Aging Study of Japanese-American men (8.9%) [4]. However, the Mayo Clinic Study of Aging (age ≥ 70 years, 72.1% male) revealed a much higher prevalence of EDS (22.3%) [6]. The differences in study participants’ characteristics (e.g., age, race, sex, education, and residential areas) may be partially responsible for the different prevalences of EDS across studies.

Our study showed a decreased prevalence of EDS along with increasing age, aligning with the results from meta-analysis and several population-based studies [7]. We found that EDS was more common in males than in females, which was consistent with previous reports [3, 8]. Epidemiological studies have indicated that OSA is a strong risk factor of EDS, and the prevalence of OSA was significantly higher in males than in females [9]. Moreover, EDS is related to increased risk of mortality, suggesting selective survival bias may be subject to the observed cross-sectional associations [10].

We observed that hypertension was correlated with a decreased prevalence of EDS, which was in agreement with a population-based study from Lausanne, Switzerland [11]. The sympathetic nervous system may be more active in people with hypertension, which could lead to a state of heightened arousal [12], but the underlying mechanisms require further investigation.

We observed that the prevalence of EDS was associated with depressive symptoms, independent of multiple potential confounders. A cross-sectional study of Japanese-American men residing in Hawaii showed a higher prevalence of EDS in people with depressive symptoms than those without [4]. The potential mechanisms that may contribute to the association included alterations in the homeostatic regulation, circadian regulation of physiological pathways, and abnormalities in the neuroendocrine system [13].

Our study found a correlation between poor sleep quality and EDS, which was similar to the report from previous studies [2, 11]. Poor sleep quality can lead to overall insufficient sleep and EDS. Furthermore, we discovered that high OSA risk was correlated with higher prevalence of EDS. The Penn State study found an association between OSA and EDS [14]. A possible explanation could be that chronic intermittent hypoxia and fragmented sleep of OSA can cause oxidative damage, neuronal damage, and cell loss in wake-promoting brain regions, which could eventually lead to EDS [15].

A major strength of our study is the large sample of older adults from a Chinese rural community. Furthermore, the EDS condition was subjectively evaluated using the ESS, a widely recognized and validated questionnaire commonly employed in both clinical and research settings. Several limitations are inherent in this study. First, EDS and the variables analyzed cannot be causally related due to the cross-sectional nature of the study. Second, some factors (e.g., lifestyle factors, health history, and use of medications) relied on self-reported data, which could lead to information bias. Third, although multiple potential confounding factors were controlled for in our study, residual confounding effect may still play a part owing to the lack of some unknown or unmeasurable confounding variables (e.g., social status and fatigue) [3, 8].

In summary, our population-based study found that EDS affected nearly one-tenth of Chinese elderly living in rural areas. In addition, the decreased likelihood of EDS was associated with older age, female sex, and hypertension; while the increased likelihood of EDS was related to depressive symptoms, high OSA risk, and poor sleep quality. Future longitudinal studies should explore clinically manageable factors and modifiable lifestyle factors of EDS, which may help develop the preventive and treatment interventions.

### Supplementary Information

Below is the link to the electronic supplementary material.

Supplementary file1 (DOCX 40 KB)

## Data Availability

The datasets used and/or analyzed during the current study are available from the corresponding author upon reasonable request.
