# Supplementary material for: Prevalence and associated factors of excessive daytime sleepiness in rural older adults: a population-based study
Source: Sleep Breath. 2024 Feb 19;28(3):1459–64. doi: 10.1007/s11325-024-03004-5 (PMC11196351; doi:10.1007/s11325-024-03004-5)
Supplement: Supplementary file 1 — Supplementary file1 (DOCX 40 KB) [file 11325_2024_3004_MOESM1_ESM.docx]

**Supplementary materials**

**Prevalence and associated factors of** **excessive daytime sleepiness in rural older adults: a population-based study**

Juan Ren^1^, Rui Liu^2,3^, Tong Zhao^1^, Jie Lu^2^, Cuicui Liu^1,2,4,5^, Tingting Hou^1,2,4,5^, Yongxiang Wang^1,2,4,5,6,7^, Lin Cong^1,2 4,5^, Yifeng Du^1,2,4,5,6,*^, Shi Tang^1,2,4,5,*^, Chengxuan Qiu^2,6,7^

**Author Affiliations:**

1. Department of Neurology, Shandong Provincial Hospital, Shandong University, Jinan, Shandong, P.R. China

2. Department of Neurology, Shandong Provincial Hospital Affiliated to Shandong First Medical University, Jinan, Shandong, P.R. China

3. Department of Ultrasound, Shandong Provincial Hospital Affiliated to Shandong First Medical University, Jinan, Shandong, P.R. China

4. Shandong Provincial Clinical Research Center for Neurological Diseases, Jinan, Shandong, P.R. China

5. Medical Science and Technology Innovation Center, Shandong First Medical University & Shandong Academy of Medical Sciences, Jinan, Shandong, P.R. China

6. Institute of Brain Science and Brain-Inspired Research, Shandong First Medical University & Shandong Academy of Medical Sciences, Jinan, Shandong, P.R. China

7. Aging Research Center and Center for Alzheimer Research, Department of Neurobiology, Care Sciences and Society, Karolinska Institutet-Stockholm University, Stockholm, Sweden

^*^**Corresponding Authors**: Yifeng Du, MD, PhD. E-mail: du-yifeng@hotmail.com. Shi Tang, E-mail: tangshi@sdfmu.edu.cn.

**Contents**

**Supplementary Methods**

**Supplementary Table 1.** Characteristics of the study participants.

**Supplementary Methods**

**Data Collection**

Following a structured questionnaire, data were collected by trained staff through face-to-face interviews, clinical examinations, neuropsychological tests, and laboratory tests in March-September 2018, as previously reported[1-3]. The questionnaire included sociodemographic factors (e.g., age, sex, and education), lifestyle factors (e.g., smoking, alcohol consumption, and leisure-time physical activity), health conditions (e.g., hypertension, diabetes, and coronary heart disease [CHD]), depressive symptoms, obstructive sleep apnea [OSA] risk, use of medications (e.g., hypnotics, antihypertensive, and hypoglycaemic agents), and sleep characteristics. All medications were classified and coded according to the Anatomical Therapeutic Chemical (ATC) classification system, as previously reported[1]. Weight and height were measured with participants wearing light clothes without shoes. Sitting arterial blood pressure was measured on the right arm using an electronic sphygmomanometer (HEM-7127J, Omron Corporation, Kyoto, Japan) after at least a 5-min rest. The 12-lead resting electrocardiogram (ECG) was recorded by an electrocardiograph (CM300, COMEN, Shenzhen, China) and then analyzed by a physician. Peripheral blood samples were taken after an overnight fast. Blood glucose, total cholesterol (TC), triglycerides (TG), low-density lipoprotein cholesterol (LDL-C), and high-density lipoprotein cholesterol (HDL-C) were measured using an Automatic Biochemical Analyzer (CS-600B, DIRUI Corporation, Changchun, China) at the Yanlou Town Hospital laboratory.

**Assessment of Covariates**

Body mass index (BMI) was calculated as weight in kilograms divided by the square of height in meters. Education was divided into illiteracy (no schooling education), primary school (1-5 years), and middle school and above (≥6 years). Leisure-time physical activity was defined as doing any type of physical activity during leisure time at least once a week. We categorized smoking status and alcohol consumption as never, former, and current smoking or drinking alcohol. Hypertension was defined as having blood pressure ≥140/90 mm Hg or current use of antihypertensive drugs (ATC codes C02, C03, and C07- C09). Diabetes mellitus was defined as having a self-reported history of diabetes, or fasting blood glucose level ≥7.0 mmol/L, or use of glucose-lowering drugs, or use of insulin injection (ATC code A10). Dyslipidemia was defined as TC ≥6.22 mmol/L, or TG ≥2.27 mmol/L, or LDL-C ≥4.14 mmol/L, or HDL-C <1.04 mmol/L, or use of hypolipidemic agents (ATC code C10)[2]. CHD was defined according to self-reported history or ECG examination, including angina, myocardial infarction, coronary angioplasty, and coronary artery bypass grafting. Stroke was defined according to self-reported history and neurological examination. The 15-item Geriatric Depression Scale (GDS-15) was used to assess depressive symptoms, and the presence of depressive symptoms was defined as a total GDS-15 score ≥5[4]. Hypnotics included hypnotics and sedatives (ATC code N05C). The sleep duration (hours/night) was categorized as short (≤6), normal (>6 to 8, reference), and long (>8) sleep duration. We used the Berlin questionnaire (BQ) to assess the OSA risk. BQ consists of three categories of symptoms, which could classify individuals into having a high risk of OSA if two or more categories had positive score and low risk of OSA if only one or no category had positive score[5]. The Pittsburgh Sleep Quality Index (PSQI) was used to assess the sleep quality[6]. Poor sleep quality was defined as a PSQI score >7[7].

**References**

**1.** Cong L, Ren Y, Hou T, et al(2020) Use of Cardiovascular Drugs for Primary and Secondary Prevention of Cardiovascular Disease Among Rural-Dwelling Older Chinese Adults. *Frontiers in Pharmacology* 11. <https://doi.org/10.3389/fphar.2020.608136>

**2.** Han X, Jiang Z, Li Y, et al(2021) Sex disparities in cardiovascular health metrics among rural-dwelling older adults in China: a population-based study. *BMC Geriatr* 21(1):158. <https://doi.org/10.1186/s12877-021-02116-x>

**3.** Wang Y, Han X, Zhang X, et al(2022) Health status and risk profiles for brain aging of rural-dwelling older adults: Data from the interdisciplinary baseline assessments in MIND-China. *Alzheimers Dement (N Y)* 8(1):e12254. <https://doi.org/10.1002/trc2.12254>

**4.** Underwood M, Lamb SE, Eldridge S, et al(2013) Exercise for depression in elderly residents of care homes: a cluster-randomised controlled trial. *Lancet* 382(9886):41-49. <https://doi.org/10.1016/S0140-6736(13)60649-2>

**5.** Chiu HY, Chen PY, Chuang LP, et al(2017) Diagnostic accuracy of the Berlin questionnaire, STOP-BANG, STOP, and Epworth sleepiness scale in detecting obstructive sleep apnea: A bivariate meta-analysis. *Sleep Medicine Reviews*:S1087079216301277. <https://doi.org/10.1016/j.smrv.2016.10.004>

**6.** Buysse DJ, Rd C, Monk TH, et al(1989) The Pittsburgh Sleep Quality Index: a new instrument for psychiatric practice and research. *Psychiatry research* 28. <https://doi.org/10.1016/0165-1781(89)90047-4>

**7.** Li J, Yao YS, Dong Q, et al(2012) Characterization and factors with sleep quality among rural elderly in China. *Archives of gerontology and geriatrics* 56(1). <https://doi.org/10.1016/j.archger.2012.08.002>

**Supplementary Table 1.** Characteristics of the study participants.

| Characteristics^a^ | Total sample (n=4845) | Excessive daytime sleepiness | | |
| --- | --- | --- | --- | --- |
|  |  | No (n=4395) | Yes (n=450) | *P* |
| Age (years), mean (SD) | 70.3 (5.0) | 71.4 (5.0) | 70.4 (4.6) | **<0.001** |
| Female, n (%) | 2774 (57.3) | 2543 (57.9) | 231 (51.3) | **0.008** |
| Educational level, n (%) |  |  |  | 0.879 |
| Illiteracy | 1888 (39.0) | 1708 (38.9) | 180 (40.0) |  |
| Elementary school | 2154 (44.5) | 1956 (44.5) | 198 (44.0) |  |
| Middle school or above | 803 (16.6) | 731 (16.6) | 72 (16.0) |  |
| BMI (kg/m^2^), mean (SD) | 24.90 (3.78) | 24.87 (3.77) | 25.26 (3.82) | **0.048** |
| Alcohol consumption, n (%) |  |  |  | **0.011** |
| Never | 2970 (61.3) | 2712 (61.7) | 258 (57.3) |  |
| Former | 460 (9.5) | 400 (9.1) | 60 (13.3) |  |
| Current | 1415 (29.2) | 1283 (29.2) | 132 (29.3) |  |
| Smoking, n (%) |  |  |  | 0.062 |
| Never | 3118 (64.4) | 2846 (64.8) | 272 (60.4) |  |
| Former | 999 (20.6) | 905 (20.6) | 94 (20.9) |  |
| Current | 728 (15.0) | 644 (14.7) | 84 (18.7) |  |
| Leisure-time physical activities, n (%) | 3236 (66.8) | 2956 (67.3) | 280 (62.2) | **0.030** |
| Hypertension, n (%) | 3216 (66.9) | 2930 (67.2) | 286 (64.0) | 0.168 |
| Diabetes, n (%) | 701 (14.5) | 623 (14.2) | 78 (17.3) | 0.070 |
| Dyslipidemia, n (%) | 1152 (23.8) | 1046 (23.8) | 106 (23.6) | 0.908 |
| CHD, n (%) | 1061 (21.9) | 1952 (21.7) | 109 (24.2) | 0.211 |
| Stroke, n (%) | 765 (15.8) | 667 (15.2) | 98 (21.8) | **<0.001** |
| Depressive symptoms, n (%) | 506 (10.6) | 389 (9.0) | 117 (26.5) | **<0.001** |
| Hypnotics use, n (%) | 240 (5.0) | 214 (4.9) | 26 (5.8) | 0.399 |
| Sleep duration (h), mean (SD) | 6.62 (1.72) | 6.64 (1.70) | 6.40 (1.94) | **0.008** |
| OSA risk, n (%) |  |  |  | **<0.001** |
| High | 1426 (29.4) | 1213 (27.6) | 213 (47.3) |  |
| Low | 3413 (70.4) | 3176 (72.3) | 237 (52.7) |  |
| ESS, mean (SD) | 4.47 (4.27) | 3.46 (2.84) | 14.34 (3.24) | **<0.001** |
| PSQI, mean (SD) | 6.02 (4.21) | 5.84 (4.17) | 7.85 (4.17) | **<0.001** |

Abbreviations: EDS, excessive daytime sleepiness; BMI, body mass index; CHD, coronary heart disease; OSA, obstructive sleep apnea; ESS, Epworth Sleepiness Scale; PSQI, Pittsburgh Sleep Quality Index Scale.

^a^ Numbers of total participants with missing values were 25 for BMI, 1 for leisure-time physical activities, 38 for hypertension, 76 for depressive symptoms, 13 for hypnotics use, 33 for sleep duration, 6 for OSA risk, and 33 for PSQI. In subsequent analyses, a dummy variable was created for participants with missing data in each of the covariates.

**Abbreviations**

BMI, body mass index

BQ, Berlin questionnaire

CHD, coronary heart disease

ECG, electrocardiogram

EDS, excessive daytime sleepiness

ESS, Epworth Sleepiness Score

GDS-15, The 15-item Geriatric Depression Scale

HDL-C, high-density lipoprotein cholesterol

LDL-C, low-density lipoprotein cholesterol

MIND-China, Multimodal Interventions to Delay Dementia and Disability in Rural China

PSQI, Pittsburgh Sleep Quality Index

OSA, obstructive sleep apnea

TC, total cholesterol

TG, triglyceride
